# Water Poultices

**Published:** 1885-02

**Authors:** 


### WATER POULTICES.

A water poultice for the throat may be made as follows: Take four thicknesses of old cotton cloth, three or four inches wide, and long enough to extend from ear to ear. Dip it in water, hot or cold, as the throat may require it. Over these folds of cotton cloth apply a layer of cotton-batting, an inch in thickness, and long and wide enough to cover the cotton cloth. Over this place a strip of waterproof cloth, or oil-silk. Apply to the throat, and keep in place by a bandage.

This poultice, if cold, will induce a local sweating, that will relieve the mucous membrane, or inner lining of the throat. When nearly dry, wring the four folds of cotton cloth again in cold water, and repeat the application for two or four nights, if necessary.

It may be wise to keep children, who are thus treated, within the house for a few days; but if they must go out on the following morning, wash the throat with cold water just before the patient leaves the house.

The water poultice should be cold in all cases in which inflammation may exist, but should be hot if ulceration or suppuration exists. A *sore* throat needs cold water, but suppuration needs hot,—as the diseased throat of diphtheria, or scarlatina in abscesses.

A sponge poultice has some advantages above all others, and is made as follows: Make a flannel bag three inches wide, and as long as any given affection requires. Fill this bag with very small bits of sponge; then soak in hot or cold water, as the case may need. Cover the outside with layers of cotton-batting, and over this some waterproof material. This poultice is elastic, and may be kept constantly near the skin, and will keep the throat at a uniform temperature,—a very important point in diphtheria and scarlatina, and all other maladies in which suppuration exists. Cold sponge poultices may be applied in the early stages of scarlatina and diphtheria to lessen the tendency to inflammation.

